# Interaction mechanism between luteoloside and corn silk glycans and the synergistic role in hypoglycemic activity

**DOI:** 10.1007/s13659-024-00428-0

**Published:** 2024-01-16

**Authors:** Shihui Qin, Yanlang Li, Huiyan Shao, Yang Yu, Yina Yang, Yi Zeng, Jia Huang, Jiang-miao Hu, Liu Yang

**Affiliations:** 1 College of Pharmacy, Anhui University of Chinese Medicine, Hefei, Anhui 230012 China; 2 State Key Laboratory of Quality Research in Chinese Medicine, Macau Institute for Applied Research in Medicine and Health, Macau University of Science and Technology, Taipa, Macau 999078 China; 3 State Key Laboratory of Phytochemistry and Plant Resources in West China, Yunnan Key Laboratory of Natural Medicinal Chemistry, Kunming Institute of Botany, Chinese Academy of Sciences, Kunming, Yunnan 650201 China

**Keywords:** Corn silk glycans, CSGs/LUT complexes, The molecular interaction mechanism, The synergistic role

## Abstract

As the two most principal active substances in the corn silk, polysaccharides and flavonoids, the mechanism of interaction between them has been a topic of intense research. This study provides an in-depth investigation of the interaction mechanism between corn silk glycans and luteoloside (LUT) and the synergistic role that result from this interaction. The interaction mechanism was evaluated by isothermal titration calorimetry (ITC) and circular dichroism (CD), and the synergistic role was evaluated by the expression of glucose transporters (GLUT-1), insulin secretion and surface plasmon resonance (SPR). CD and ITC results indicated that the interaction between CSGs and LUT mainly driven by the Cotton effects, enthalpy and entropy-driven. This interaction precipitated the formation of complexes (CSGs/LUT complexes) between corn silk glycans (CSGs) with four different molecular weights and luteoloside (LUT). Furthermore, the CSGs and LUT play a synergistic role in glucose regulation through GLUT-1 expression and insulin secretion experiments, compared to single luteoloside group.

**Graphical Abstract:**

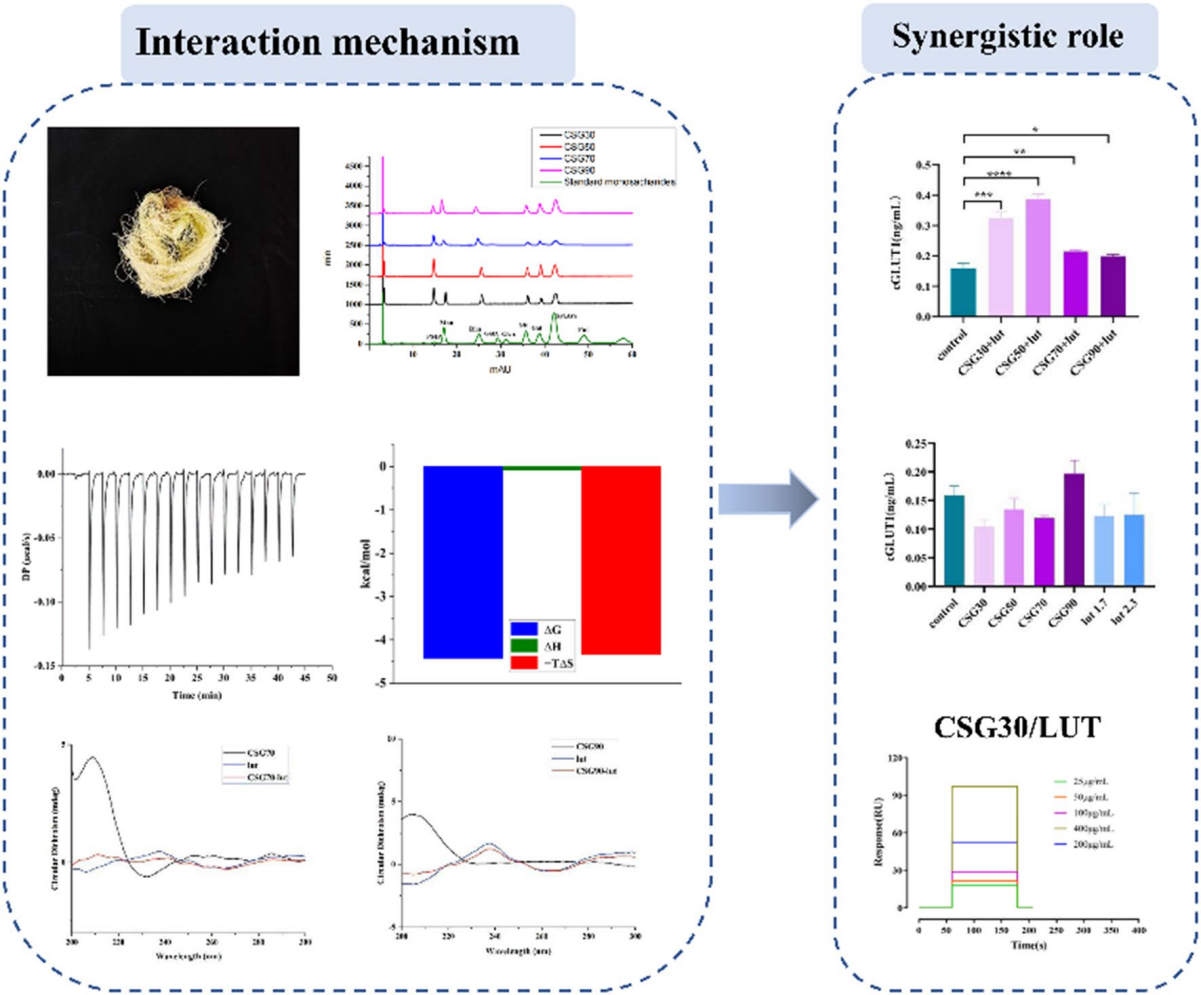

**Supplementary Information:**

The online version contains supplementary material available at 10.1007/s13659-024-00428-0.

## Introduction

Glycans in food systems could interact with co-existing small molecules and proteins normally during their absorption, digestion and biological process [1]. This complex coacervation behavior can affect the texture, structure, and stability of food [2, 3]. These coacervates result from spontaneous mechanisms coming from interactions [4]. Several types of forces can prompt the formation of this complex structure such as electrostatic, hydrophobic, hydrophilicity, and van der Waals [5, 6]. Glycans are building the cornerstone of complex coacervation, at micro- and nano-scales [7–9]. Various investigations have shown that conglomeration can affect biological activities. Ginseng polysaccharides can co-exist with small molecules and then play a synergistic role in the regulation of gut microbiota [10]. The complex coming from with *moringa oleifera* leaf flavonoids and polysaccharides can possess a stronger hypoglycemic/hypolipemic potential [11].

During centuries of empirical clinical use, traditional Chinese medicines (TCM) are mostly prepared water decoctions for prophylactic and therapeutic diseases. Unlike Western medicines, polysaccharides, proteins and small molecules are the three dominant chemicals in TCM decoction [10]. However, the interactions between small molecules and glycans, their synergistic roles, and which parts contribute to therapeutic effects in TCM decoctions remain unknown. Corn silk is a common TCM that people usually take after boiling to achieve the effect of hypoglycemic. Corn silk is rich in flavonoids [12], polysaccharides [13], terpenoids [14] and so on. Corn silk extract has been reported to have hypoglycemia [15], antioxidation [16], antitumor [17], antibacterial [18] and other pharmacological activities. Researchers mostly use the method of first decolorization and then mellow precipitation when extracting polysaccharides [19]. Guo et al. used analytical kinetic simulations to characterize the interaction between polysaccharides and flavonoids, thereby improving the activity of polysaccharides on α-amylase and α-glucosidase [20].

In order to restore the actual decocting method of corn silk, this study first directly extracted corn silk with water and graded alcohol precipitation to obtain four different total extracts of water decoction (CSGC), and analyzed their main substance composition. Subsequently, CSGC underwent decolorization and preliminary purification to obtain four colorless and protein free corn silk crude glycans (CSGs). Isothermal titration calorimetry (ITC) and circular dichroism (CD) be employed to analyze the interaction principle between CSGs and flavonoids (luteoloside, LUT), and investigate the synergistic role in the regulation of blood sugar. Polysaccharides and flavones coexist in the food system, and the mechanism of their deep interaction remains to be solved. Simultaneously, elucidation of these issues would be significant for the modernization of TCM and functional food.

## Results and discussion

### Structural characterization of CSGC and CSGs

High performance gel permeation chromatography (HPGPC) was used to analyze the molecular weight and purity of CSGC and CSGs (Additional file SI 1: Fig. S1, S2).The content of total sugar, protein, flavonoid, uronic acid and Mw composition of CSGC and CSGs were summarized in Table 1. The total sugar content of CSGC was lower than that of CSGs, the flavonoid and uronic acid content of CSGC were higher than that of CSGs, and the molecular weight was also different.

**Table 1:** Composition and molecular weight analysis of CSGC and CSGs

Sample	Total sugar composition (%, w/w)	Protein (%, w/w)	Total flavonoid (%, w/w)	Uronic acid (%, w/w)	Mw (Da)
CSGC30	69.88	19.14	8.1454	11.71	5.81 × 10^7^; 8.52 × 10^6^; 3.22 × 10^5^; 436
CSGC50	59.22	16.29	8.9599	6.62	8.87 × 10^6^; 5.03 × 10^5^; 436
CSGC70	50.21	24.52	7.2979	5.47	6.29 × 10^6^; 2.64 × 10^5^; 436
CSGC90	48.45	18.21	8.4169	4.48	3.39 × 10^4^; 436
CSG30	72.77	19.08	1.1636	7.93	4.9 × 10^3^
CSG50	67.48	15.57	3.8739	6.41	2.91 × 10^5^; 9.28 × 10^3^
CSG70	73.15	14.19	1.9394	2.01	3.0 × 10^5^; 6.14 × 10^3^
CSG90	72.64	16.02	1.5127	4.83	3.14 × 10^5^; 5.58 × 10^3^

### Assay for monosaccharide composition

**Fig. 1:**
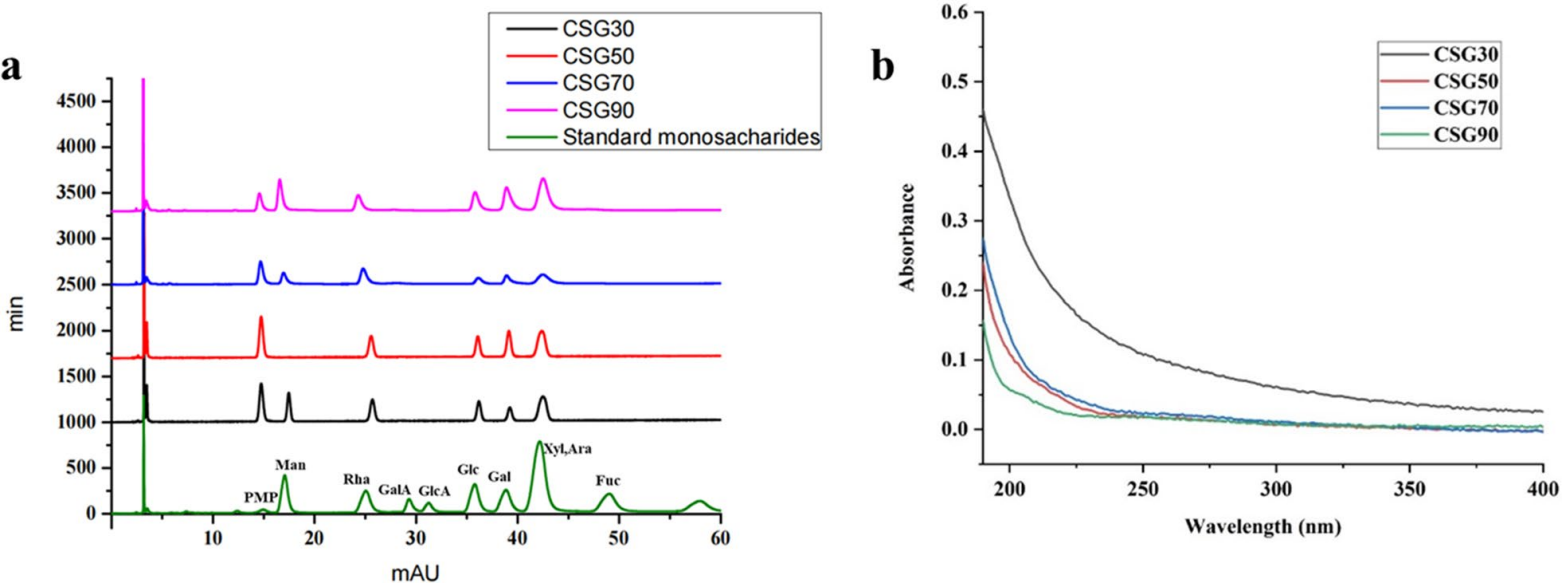
Primary structural features of CSGs. **a** Monosaccharide composition of CSGs. **b** UV spectrum of CSGs

The monosaccharide composition of CSGs was analyzed by PMP derivation method (Fig. 1a). The results showed that CSG30, CSG70, and CSG90 were composed of mannose, rhamnose, glucose, galactose, and arabinose, but with different mole ratio, respectively: 0.21:0.21:0.13:0.19:0.25; 0.15, 0.28, 0.11, 0.15, 0.30; 0.17: 0.12: 0.14: 0.19: 0.37,0.37. CSG50 was deficient in the mannose, with the molar ratio of 0.20: 0.17: 0.22: 0.39. As displayed in the UV spectrum (Fig. 1b), CSGs had no absorption peak from 190 to 400 nm, which confirmed that CSGs did not contain proteins and nucleic acids.

### FT-IR spectrum of CSGs

**Fig. 2:**
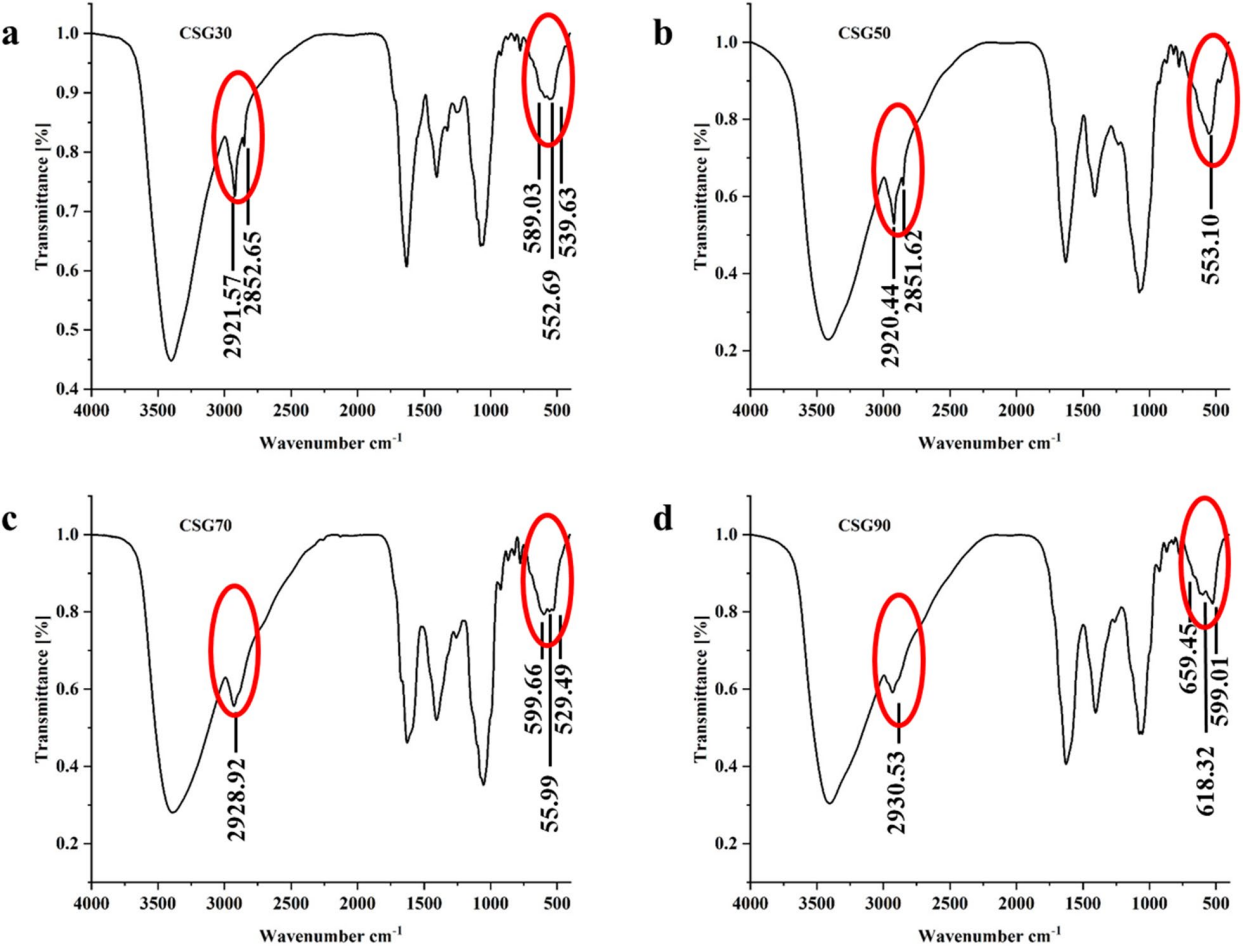
Primary structural features of CSGs. **a** IR spectrum of CSG30. **b** IR spectrum of CSG50. **c** IR spectrum of CSG70. **d** IR spectrum of CSG90

Comparing the four FT-IR spectra (Fig. 2), it could be detected that the difference between CSG30, 50 and CSG70, 90 was mainly due to the vibration absorption peak at 2850 cm^−1^. The divide between CSG 50 and the other three spectrograms was the absorption peak near 599 cm^−1^ in the fingerprint area. The vibration absorption peak at 2920–2850 cm^−1^ owed to the stretching vibration of –CH_3_, –CH_2_, and –CH [21]. The absorption peak from 1600 cm^−1^ to 1900 cm^−1^ attributed to the stretching.

vibration of –C=O [22, 23]. The characteristic peaks in the fingerprint area (400–1000 cm^−1^) could be used to distinguish the types of monosaccharides [24]. The deletion of the absorption peak near 600 cm^−1^ in CSG50 FT-IR spectra (Fig. 2b) indicated that CSG50 did not contain mannose, which was consistent with the results of CSGs monosaccharide composition analysis. CSG30, CSG50, CSG70, and CSG90 had absorbed around 1625 cm^−1^, suggesting that all of the CSGs contain uronic acid, which was consistent with the results of uronic acid content.

### Interactions between luteoloside and CSGs

#### CD spectra analysis of LUT and CSGs/LUT complexes

**Fig. 3:**
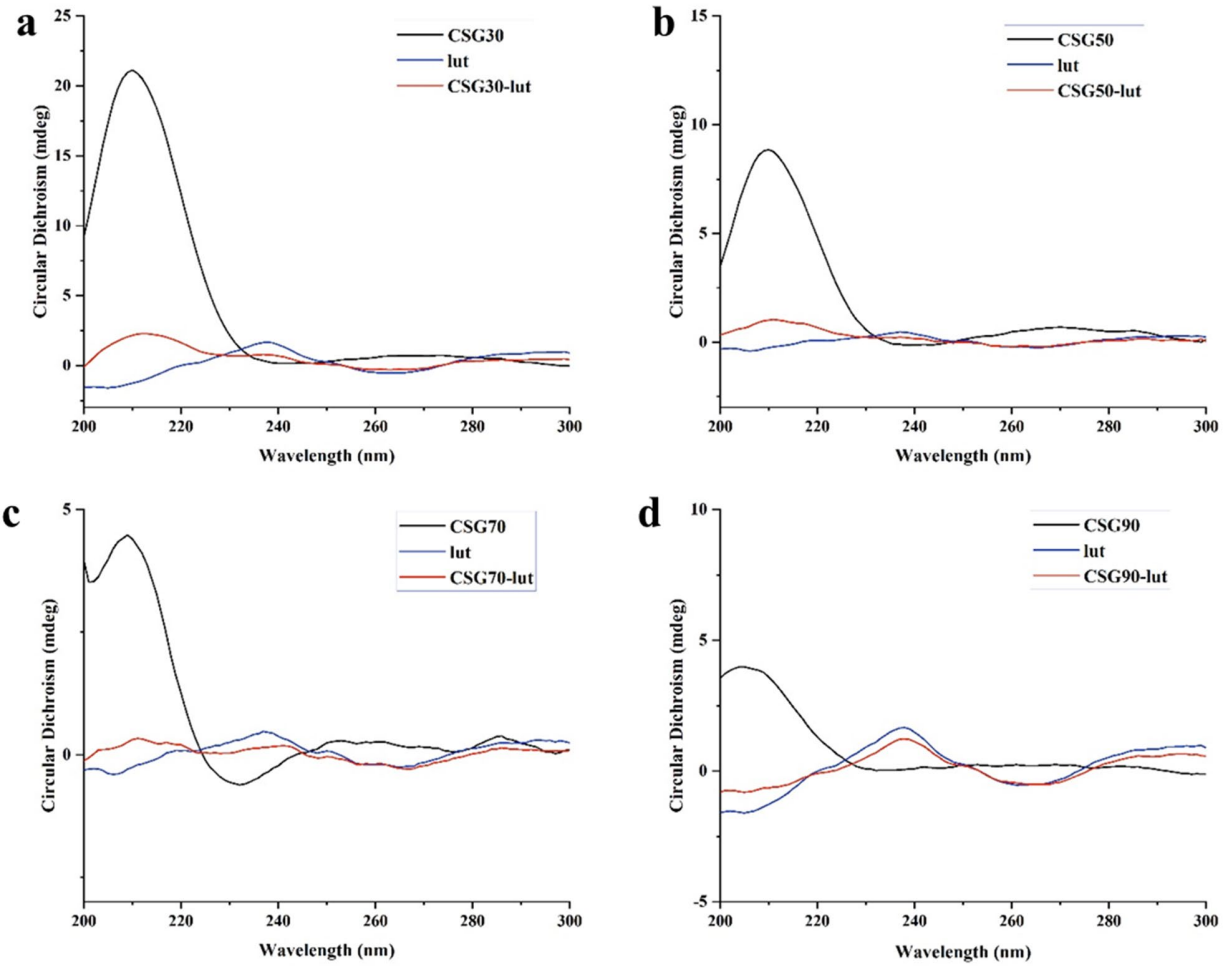
The interaction of CSGs/LUT complexes were identified by circular dichroism spectroscopy (CD). **a** CSG30 (5 mg/mL, 40 µL), LUT (3 mg/mL, 260 µL), and CSG30/LUT complexes. **b** CSG50 (5 mg/mL, 40 µL), LUT (2 mg/mL, 260 µL), and CSG50/LUT complexes. **c** CSG70 (5 mg/mL, 40 µL), LUT (2 mg/mL, 260 µL), and CSG70/LUT complexes. **d** CSG90 (2 mg/mL, 40 µL), LUT (3 mg/mL, 260 µL), and CSG90/LUT complexes

When the molecule contains several chromogenic groups, the CD spectrum can effectively distinguish the positive and negative Cotton effects (CEs) of each absorption band. Carbonyl compounds can emanate two main transitions due to the influence of other functional groups (electronegativity of oxygen and conjugated double bonds): strong absorption (200–260 nm) of π → π^*^ and weak absorption of n → π^*^ around 300 nm [25]. The content of uronic acid in CSGs is about 5.00%. In the structure of LUT has α, β-unsaturated cyclic ketone. When these two chromophore groups are located closer in space, the CEs occur. The typical absorption bands of carboxylic acid derivatives and α, β-unsaturated cyclic ketone appears at 200 and 240 nm of CSGs groups and LUT group in CD spectra. However, after mixing the LUT to CSGs, the complex groups signals resulted in the CD spectra (Fig. 3). A positive CEs of the complex groups can be seen from 200 to 220 nm regions indicating that this complex was ordered. In addition, negative CEs appeared at 240 nm, suggesting that LUT may be wrapped by CSGs in water solution. The CD spectra of CSGs, LUT, and the mixed group were different evidenced that the mixing of LUT and CSGs forms a new hybrid embodiment [26].

#### ITC analysis of LUT and CSGs/LUT complexes

**Fig. 4:**
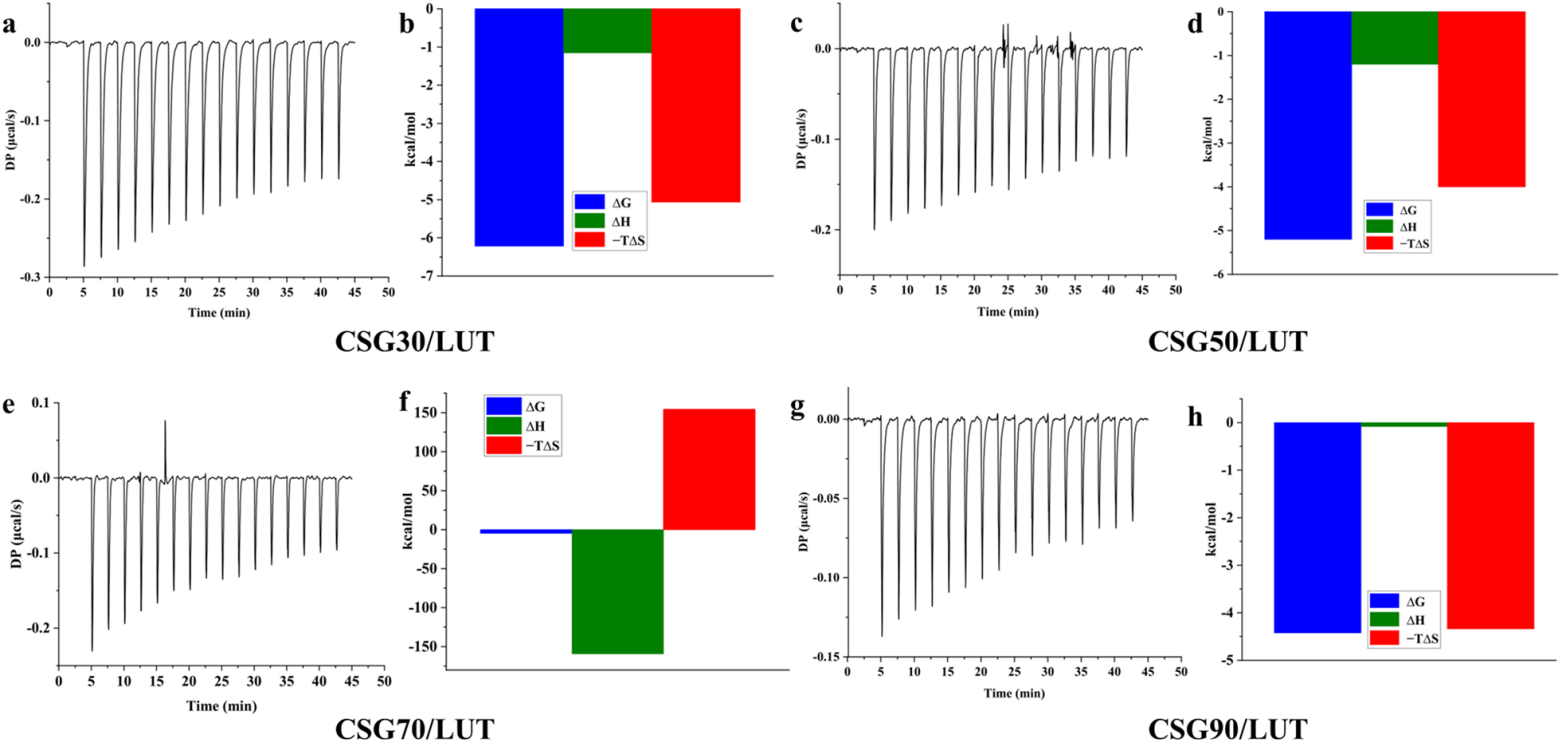
The interaction mechanism of LUT and CSGs. **a–h** The schematic diagram for CSGs/LUT complexes. **a** Standard calorimetric titrations of 5 mg/mL CSG30 into 3 mg/mL LUT solution at 25 ℃. **b** The fitting data of ITC from LUT/CSG30 complexes ΔG, ΔH, −TΔS represented Gibbs free energy of binding (blue), enthalpy changes (green), and entropy changes (red). **c** The final figure of 5 mg/mL CSG50 into 2 mg/mL LUT solution at 25 ℃. **d** The signature of ITC from LUT/CSG50 complexes. **e** Standard calorimetric titrations of 5 mg/mL CSG70 into 2 mg/mL LUT solution at 25 ℃. **f** The fitting data of ITC from LUT/CSG70 complexes. **g** The final figure of 2 mg/mL CSG90 into 3 mg/mL LUT solution at 25 ℃. **h** The signature of ITC from LUT/CSG90 complexes

The interaction mechanism between CSGs and LUT was determined by isothermal titration calorimetry (ITC). Titration with a deionized aqueous solution was used as a blank control (Additional file SI 1: Fig. S3). By titrating the CSGs aqua into the LUT liquor completed, the critical thermodynamic parameters of the equilibrium dissociation constant (KD), binding stoichiometry (N), Gibbs free energy (ΔG), entropy change (-TΔS), and enthalpy change (ΔH) were detected by software analysis. Firstly, the KD value reflected the affinity between LUT and CSGs (Table 2). The result demonstrated that the interactions between LUT and CSG30 are stronger than that in LUT/CSG50, 70, and 90 complexes. Secondly, the negative (ΔG < 0) Gibbs energy measured that the formations of the LUT/CSGs were a spontaneous interaction. Finally, according to the value of N, we calculated that CSG30 (N = 2.0 × 10^−3^), CSG50 (N = 3.7 × 10^−3^), CSG70 (N = 2.6 × 10^−5^), and CSG90 (N = 0.139), approximately bind with 500, 270.3, 38461.5, and 7.2 molecules of LUT in the solution (Fig. 4i).

We investigated the dominant intermolecular force to drive the assembly between LUT and CSGs. The signature of LUT and CSG30, 50, and 90 (Fig. 4b, d, h) showed that the ΔH values were negative (− 1.15, − 1.20, and − 8.3 kcal/mol) and the values of − TΔS were negative. This result indicated that the co-assembly between LUT and CSG30, 50, and 90 were enthalpy and entropy driven primarily by hydrogen bonds, hydrophobic effect, van der Waals force, conformational, etc. However, the entropically (− TΔS) driven by conformational and hydrophobic effect could be the hazardous factors in the LUT and CSG70 co-assembly systems (Fig. 4f). The ΔH value of the LUT and CSG70 co-assembly systems was − 159 kcal/mol suggested that the formation of CSG70/LUT was only driven by enthalpy (Additional file SI 1).

**Table 2:** Thermodynamic parameters of interactions between CSGs and luteoloside (LUT) measured by isothermal titration microcalorimetry (ITC)

LUT	N	KD (M)	ΔH (kJ/mol)	ΔG (kJ/mol)	− TΔS (kJ/mol)	Enthalpy (%)	Entropy (%)
CSG30	2.0 e^−3^	7.9 e^−6^	− 1.15	− 6.22	− 5.06	18.52	81.48
CSG50	3.7 e^−3^	1.55 e^−8^	− 1.20	− 5.20	− 4.00	23.08	76.92
CSG70	2.6 e^−5^	3.61 e^−8^	− 159	− 4.70	154	3180	− 3080
CSG90	0.139	5.74e^−8^	− 8.3	− 4.42	− 4.34	65.66	34.34

Average of duplicates for each. n: stoichiometry, KD: affinity level, ΔH: enthalpy, ΔS: entropy, ΔG: free enthalpy, T: temperature. Enthalpy (%) = ΔH/(ΔH – TΔS) ×100%; Entropy (%) = − TΔS/(ΔH –  TΔS) × 100%. Pooled SD: pooled standard deviation

#### FI-IR analysis of CSGs and CSGs/LUT complexes

**Fig. 5:**
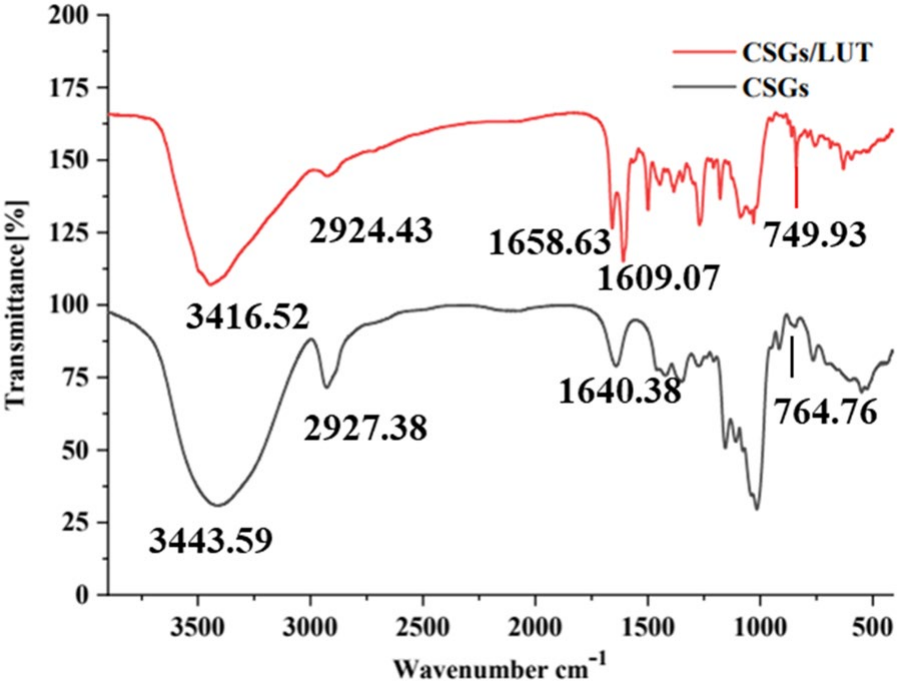
Infrared spectra of CSGs and CSGs/LUT

The combination of CSGs and LUT was characterized by infrared spectroscopy. The FI-IR spectra of CSGs and CSGs/LUT complex are shown in Fig. 5. The FT-IR spectra of CSGs/LUT after interaction are similar to those of CSGs. The broad peak intensity at 3443.59 cm^−1^ is lower than that of CSGs, possibly due to a decrease in the number of hydroxyl groups. After the interaction, the tensile vibration strength of methyl C–H (2927.38 cm^−1^) and the bending vibration strength outside the O–H plane (764.76 cm^−1^) were weakened, which may be due to the formation of hydrogen bonds between the hydroxyl group of LUT and the hydroxyl group of CSGs.

#### The expression of GLUT-1 by LUT and CSGs/LUT complexes

**Fig. 6:**
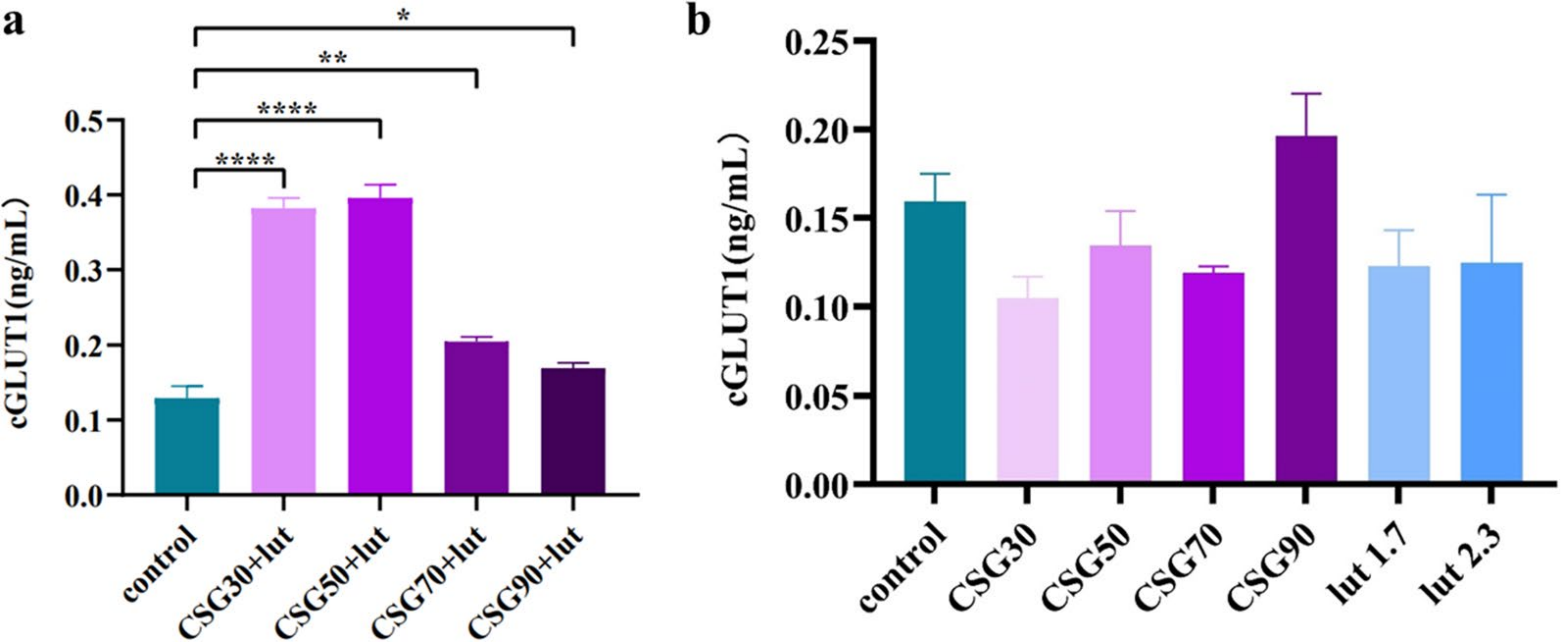
The expression of GLUT-1 in HCT116 cells after 24 h of treatment with CSGs, LUT and CSGs/LUT complexes. **a** GLUT-1 expression of CSGs/LUT complex (200 μg/mL) on HCT116 cells. **b** GLUT-1 expression of CSGs (200 μg/mL) and LUT (1.7 and 2.3 mg/mL) on HCT116 cells. Data represent the means ± standard errors of the means of at least three independent experiments; *p < 0.05, **p < 0.01, and ***p < 0.001

The changes in GLUT-1 expression can significantly alter the basal glucose uptake. When GLUT-1 expression is increased, it modulates glucose transport into cells and lowers blood sugar [27]. After treatment with LUT and CSGs/LUT complexes, the expression of GLUT-1 in HCT-116 cells was detected. The result suggested that compared with the control group, the CSGs/LUT complexes treated group dramatically raised the expression of GLUT-1 (Fig. 6a). Among them, the GLUT-1 expression level of the CSG30/LUT and CSG50/LUT treatment groups are superior to others (Fig. 6b). The investigate of GLUT-1 expression also hinted that the synergistic role between CSG30, CSG50 and LUT in hypoglycemic activity.

### Assay for direct interaction by surface plasmon resonance (SPR)

**Fig. 7:**
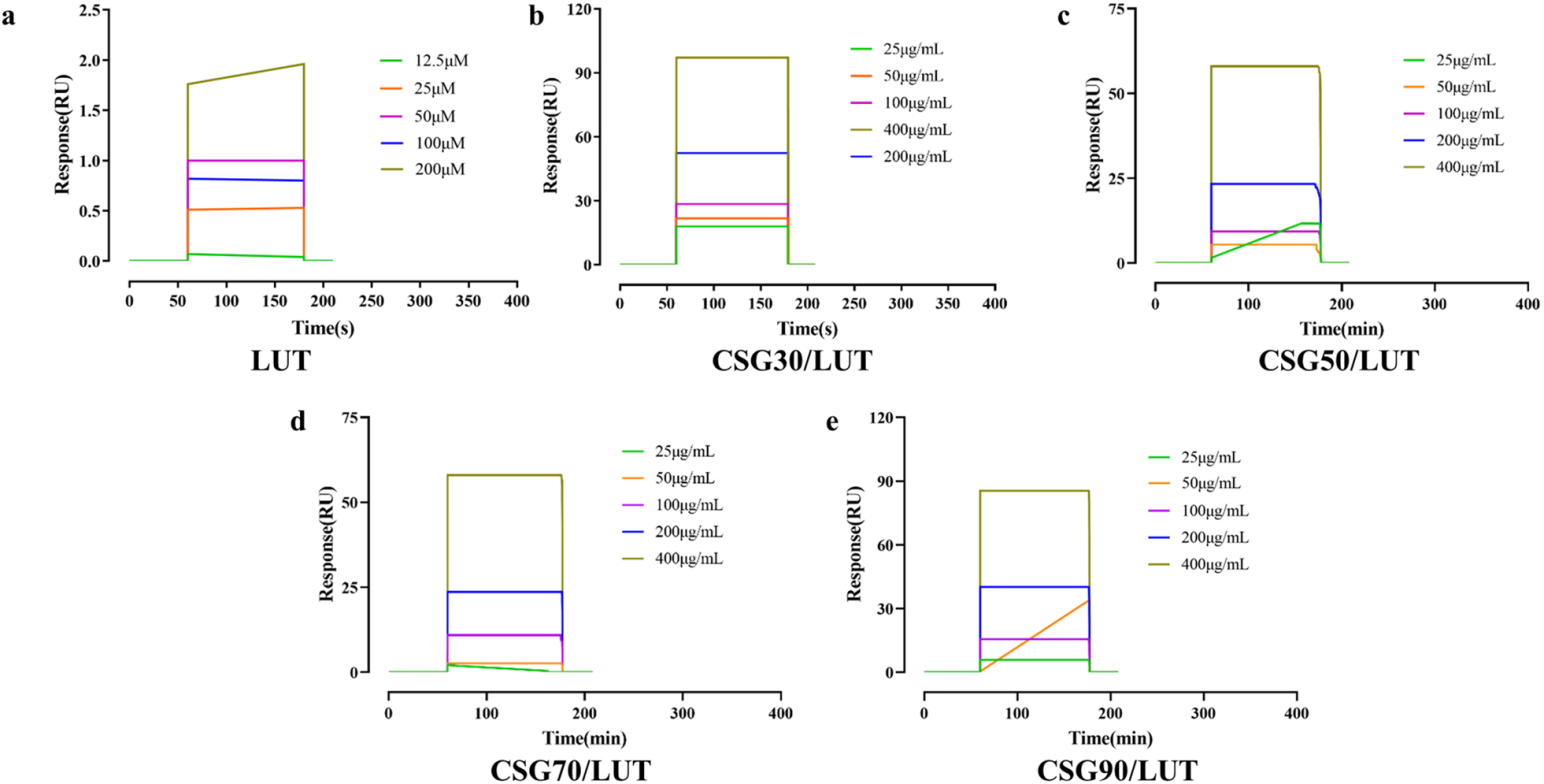
The affinity between LUT, CSGs/LUT complexes and Glut-1. **a** The sensorgram of LUT interacts with GLUT-1 protein at different concentrations. **b–e** The sensorgram of CSGs/LUT complexes interacts with GLUT-1 protein at 25, 50, 100, 200, and 400 µg/mL

To further study whether LUT and CSGs/LUT complexes from corn silk directly bind to GLUT-1 protein, we investigated the binding affinity using a SPR system. The data of SPR experimentation showed (Additional file SI 1: Table S1) that the KD values of CSGs/LUT complexes and GLUT-1 (Fig. 7b–e), were 1.70 × 10^−4^ M,1.73 × 10^−4^ M, 2.02 × 10^−4^ M, and 1.17 × 10^−2^ M, respectively. However, the affinity among LUT and GLUT-1 is only 2.2 × 10^−2^ M (Fig. 7a). The affinity of LUT/CSG30, CSG50 and CSG70 complexes on GLUT-1 protein was stronger than that of LUT/CSG90 complex.

#### The expression of insulin by LUT and CSGs/LUT complexes

**Fig. 8:**
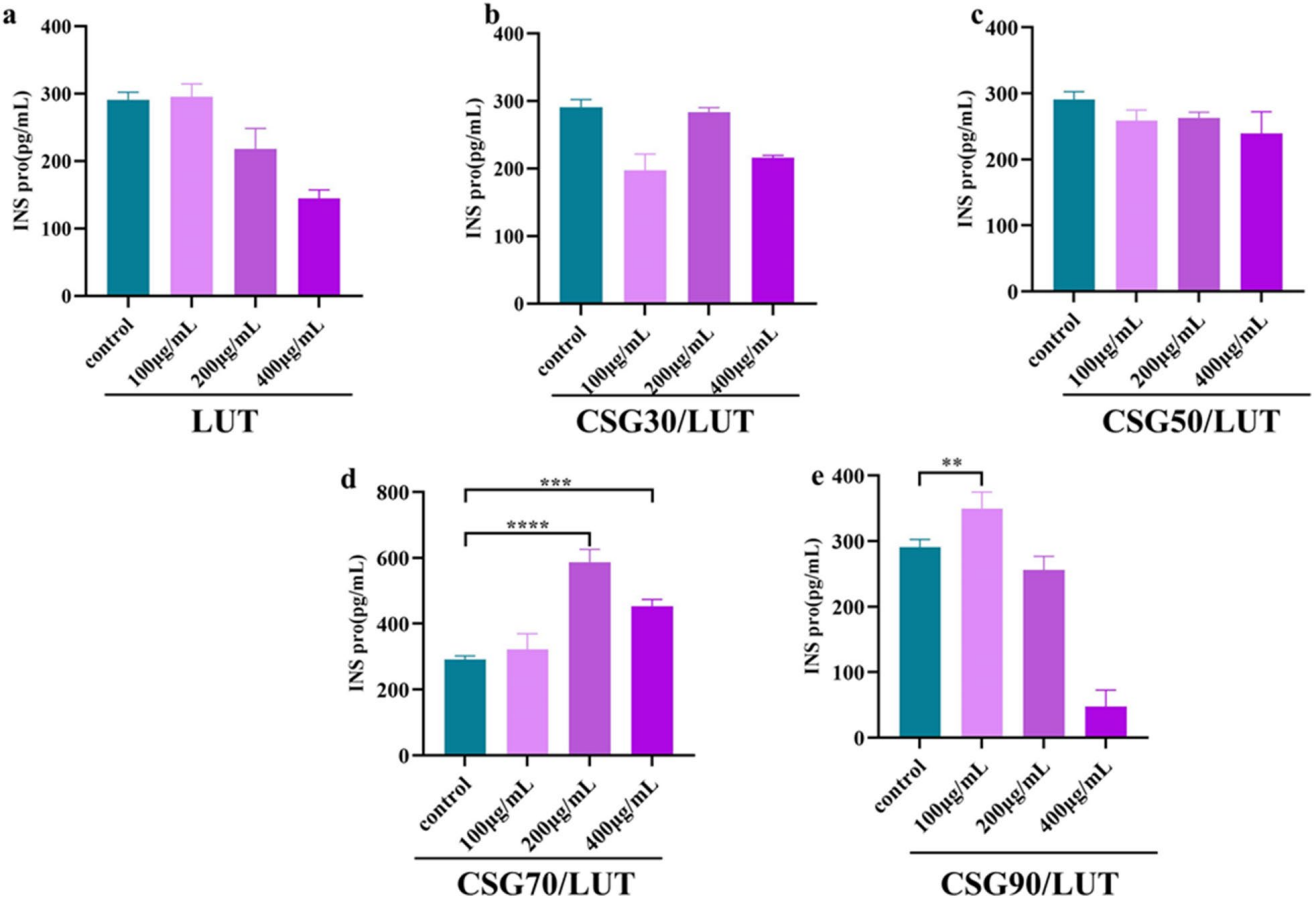
MIN6 cells were treated with LUT and CSGs/LUT complexes (100, 200, and 400 µg/mL) for 24 h. We detected the protein secretion of insulin. **a **The secretion of insulin protein was detected in MIN6 cells treated with LUT. **b-e** The secretion of insulin protein was detected in MIN6 cells treated with CSGs/LUT complexes.  Data represent the means ± standard errors of the means of at least three independent experiments; *p < 0.05, **p < 0.01, and***p < 0.001

The level of insulin directly determines the concentration of blood sugar. With CSGs, LUT and CSGs/LUT complexes after processing, the detection of MIN6 cell secretion of insulin. The results showed that the CSGs/LUT complex treatment group significantly increased the secretion of insulin compared with the control group (Fig. 8). Among them, the insulin secretion of CSG70/LUT and CSG90/LUT treatment groups was better than that of other treatment groups. Studies on insulin secretion revealed a synergistic effect between CSG70, CSG90 and LUT.

## Conclusions

In this work, the binding mechanism of glycans and luteoloside from corn silk and the synergistic role of CSGs and LUT in hypoglycemic activity. ITC and CD spectra analysis showed that LUT binding to CSGs was promptly, spontaneously, and successfully. Our results suggested that in functional food systems, the interaction between LUT and CSGs happens. Furthermore, the CD data displays that the formation of LUT/CSGs complex mainly depends on the Cotton effects of the carbonyl functional groups in the LUT and CSGs structures. The ITC data calculated that enthalpy-driven and entropy-driven (covering hydrogen bonds, coulomb force, conformation, and hydrophobic effect, et al.) played a critical role in the formation of LUT/CSGs (CSG30, CSG50, and CSG90) complex. The interaction between CSGs and LUT was analyzed by infrared spectroscopy, and the main mechanism of the complex was hydrogen bond. More interestingly, it was found that the formation of LUT/ CSGs complex improved the glucose-regulating activity of LUT through insulin secretion stimulating and GLUT-1 expression stimulating experiments. Altogether, these findings not only contribute to the understanding of the interaction mechanism between glycans and luteoloside, but also pave the way to demonstrate the practical effectiveness of functional substances in the field of functional foods.

## Experimental section

### Materials and chemicals

Corn silk was collected from the local market in Kunming, Yunnan Province, China. Diethylaminoethanol (DEAE) was from GE Healthcare (Sweden). Monosaccharide standards (Rhamnose, Fructose, Arabinose, Xylose, Mannose, Glucose, Galactose, Glucuronic acid and Galacturonic acid standards), 1-phenyl-3-methyl-5-pyrazolone (PMP) and trifluoroacetic acids (TFA) were obtained from Innochem Co. Ltd (Beijing, China). Aluminum nitrate, sodium nitrite and sodium hydroxide were bought from Damao Co. Ltd (Tianjin, China). The total protein testing kit was purchased from Nanjing Jiancheng Bioengineering Institute (Nanjing, China). GLUT-1 proteins and ELISA kits were obtained from Abcam Trading (Shanghai) Co., Ltd. (Shanghai, China). Mouse insulin ELISA kit was purchased from Fine Test^®^ (Hubei) Co., Ltd (Hubei, China). All chemicals and reagents were purchased locally and were of analytical grade.

### Extraction and purification of CSGC and CSGs

Dried corn silk (440 g) was crushed and extracted with 1:10 (w/v) distilled water for 2 times at 95 ℃ for 2 h each time. All extracts were concentrated under reduced pressure at 60 ℃, and absolute alcohol was continuously added to final concentrations of 30%, 50%, 70%, and 90% at room temperature and precipitated overnight. The protein in the extract was removed by the sevage method. These protein-free extracts were freeze-dried to obtain the corn silk glycan complex (CSGC). The stained CSGC was dissolved in distilled water and decolorized on a 2.5 × 60 cm DEAE glucose fast-flow column. The column was eluted isobaric with an aqueous solution at a flow rate of 0.5 mL/min. The eluate (20 mL/tube) was collected by an automatic collector. The eluate was concentrated, dialyzed (Da ≤ 1000) and lyophilized to obtain colorless corn silk glycans (CSGs). CSGs were concentrated, lyophilized and stored in dry containers for further analysis.

### Determination of chemical composition and molecular weight of CSGC and CSGs

The total sugar content was determined by phenol-sulfuric acid method with glucose as the standard substance [28]. Protein content was determined by the BCA method based on the biuret principle [29]. The content of flavonoids was determined by UV-VIS spectrophotometry with rutin as the standard [30]. The content of uronic acid was determined by the M-hydroxy-biphenyl method [31]. The molecular weight was determined by high performance gel permeation chromatography (HPGPC), and T-series glucan was used as the standard [32].

### Determination of monosaccharide composition and UV spectrum analysis of CSGs

The monosaccharide composition of CSGs was analyzed by the modified hydrolysis combined with PMP pre-column derivatization method [33]. 1 mL TFA (4 M) was added to the 2 mg sample, stirred magnetically at 100℃ for 6 h, concentrated under reduced pressure, and methanol was added several times to remove excess TFA. Add 1 mL distilled water to the sample to dissolve, take out 50 µL, add 50 µL NaOH (0.6 M) and 100 µL PMP-methanol (0.5 M) in turn, reaction at 70℃ for 100 min, and then add 100µL HCl (0.3 M), 1mL double steaming water and 1mL chloroform solution in turn. Remove the organic phase after mixing. Repeat three times, the water intake layer through 0.22 μm microporous filter membrane. PMP-labeled oligosaccharide was analyzed using Agilent technologies 1260 series (Agilent Co. USA) and Agilent ZORBAX SB-C18 column (250 mm × 4.6 mm). The mobile phase was NaH_2_PO_4_/Na_2_HPO_4_ buffer (pH 6.8) and acetonitrile (v/v, 83:17–81:19).

The ultraviolet spectrum of CSGs (1 mg/mL) was analyzed by Shimadzu UV-2700 UV–vis spectrophotometer (Shimadzu, Japan) in the wavelength range of 190–400 nm.

### FI-IR spectral analysis of CSGs

CSGs (1.0 mg) were ground with dry KBr powder and pressed into flakes [34]. The FI-IR spectrum was determined by Fourier transform infrared spectrophotometer (FT-IR, Nicolet iS10, Thermo Fisher Scientific Inc.), and the wave number range was 4000−400 cm^−1^.

### Interaction study

#### CD analysis of LUT and CSGs/LUT complexes

The CD spectra of CSGs and CSGs/LUT complexes were determined at 25 ℃ by CD-V100 spectrometer (Applied Photophysics, England) [26]. Added the 40 µL of CSG30 (5 mg/mL)/CSG50 (5 mg/mL) CSG70 (5 mg/mL)/CSG90 (2 mg/mL) to 260 µL of LUT (3 mg/mL)/LUT (2 mg/mL)/LUT (2 mg/mL)/LUT (3 mg/mL), and mixed to given the CSG30, 50, 70, and 90/LUT complexes, successively. The concentration of CSGs/LUT complex sample solution is based on ITC titration ratio.

#### ITC analysis of CSGs and CSGs/LUT complexes

The isothermal titration calorimeter (MicroCal PEAQ-ITC, Malvern, UK) was used for thermodynamic measurements at 298 K. CSGs (5/2 mg/mL) from a 40 µL syringe were injected into a sample cell containing 260 µL LUT (2/3 mg/mL). The titration experiment was performed 17 times with 2 µL per injection and 150 s interval between injections. The experiments were conducted at 25℃, and the sample cell solution was continuously stirred at 750 rpm. A blank experiment was performed by deionized water into water. Data were analyzed and reported using MicroCal PEAQ-ITC Analysis software, and the measured binding isotherms were fitted with a “one set of sites” fitting model to obtain enthalpy change (ΔH), free energy change (ΔG, ΔG = − RT lnKa = ΔH − TΔS), and entropy change (ΔS).

#### FI-IR analysis of CSGs and CSGs/LUT complexes

1 mg of CSGs or CSGs/LUT complex was mixed with 100 mg potassium bromide (KBr) powder, respectively, compressed into disks and infrared spectra were collected at 4000−400 cm^−1^ wave number [26]. Preparation method of CSGs/LUT complex: 40 µL 5 mg/mL CSGs aqueous solution was mixed with 260 µL 2 mg/mL LUT aqueous solution, and freeze-dried for use.

#### The expression of GLUT-1 by LUT and CSGs/LUT complexes

HCT-116 cells were used to perform stimulation GLUT-1 expression test, and GLUT-1 concentrations of CSGs/LUT complex and LUT monomer were determined to determine the influence of the sample on the expression. The cells were treated with CSGs (200 μg/mL) and LUT (1.7 and 2.3 mg/mL) respectively, and the supernatant was obtained by centrifugation after lysis. The supernatant was processed with the GLUT-1 enzyme-linked immunosorbent assay kit, and the absorbance was measured with an enzyme-labeled instrument at 450 nm, and the standard curve was drawn to calculate the concentration.

### Assay for hypoglycemic activity by surface plasmon resonance (SPR)

The affinity between GLUT-1 protein and CSGs was measured using surface plasmon resonance [SPR, Biacore S200 instrument (GE Healthcare, MA, US)]. The GLUT-1 protein solution was fixed on the surface of the Series S CM5 Sensor chip (GE Healthcare) with binding and deionization times of 120 and 150 s, respectively, and then the CSGs solution (25, 50, 100, 200, 400 µg/mL) passed through the sensor surface at a speed of 30 µL/min. The Kinetics and affinity analyses of CSCs at different concentrations were calculated by Biacore S200 evaluation software (GE Healthcare) [35].

### The secretion of insulin by LUT and CSGs/LUT complexes

The concentration of insulin after addition with CSGs/LUT complexes and LUT was determined by stimulating ins secretion with MIN6 cells to determine the effect of the sample on their secretion. The cells were treated with samples of 100, 200 and 400 µg/mL respectively to absorb the supernatant. The OD of insulin was measured at 450 nm by using insulin ELISA kit.

### Statistical analysis

The data were expressed as mean ± SD (standard deviation) of triplicate determination. Statistical significance was analyzed by one-way analysis of variation (ANOVA) and student’s test with GraphPad Prism software (GraphPad, San Diego, CA, USA). P < 0.05 was statistically significant.

### Supplementary Information

**Additional file SI 1:** **Fig. S1.** Polysaccharide (CSGC) purity by high-performance gel permeation chromatography (HPGPC) profiles. The presence of 1 mg/mL CSGC monitored by HPLC-ELSD. Chromatographic conditions: sample: 1 mg/mL; chromatographic column: Shodex OHpak-SB-804 HQ 8 mm*30 cm; mobile phase: 100% water isocratic elution; Elution time: 15 min; Injection volume: 20 μL. **Fig. S2.** Polysaccharide (CSGs) purity by high-performance gel permeation chromatography (HPGPC) profiles. The presence of 1 mg/mL CSGs monitored by HPLC-ELSD. Chromatographic conditions: sample: 1 mg/mL; chromatographic column: Shodex OHpak-SB-804 HQ 8 mm*30 cm; mobile phase: 100% water isocratic elution; Elution time: 15 min; Injection volume: 20 μL. **Fig. S3. **The interactions of aqueous solution. **Fig. S4. **Scanning electron micrographs of CSGC and CSGs: (a-3) CSGC30; (b-3) CSGC50; (c-3) CSGC70; (d-3) CSGC90; (e-3) CSG30; (f-3) CSG50; (g-3) CSG70; and (h-3) CSG90; (3, 20000×). **Table S1.** SPR analysis showed that CSGS and GLUT-1 proteins had direct binding KD values

## Data Availability

This article is licensed under a Creative Commons Attribution 4.0 International License, which permits use, sharing, adaptation, distribution and reproduction in any medium or format, as long as you give appropriate credit to the original author(s) and the source, provide a link to the Creative Commons licence, and indicate if changes were made. The images or other third party material in this article are included in the article’s Creative Commons licence, unless indicated otherwise in a credit line to the material. If material is not included in the article’s Creative Commons licence and your intended use is not permitted by statutory regulation or exceeds the permitted use, you will need to obtain permission directly from the copyright holder. To view a copy of this licence, visit https://creativecommons.org/licenses/by/4.0/.
