# Supplementary material for: Interaction mechanism between luteoloside and corn silk glycans and the synergistic role in hypoglycemic activity
Source: Nat Prod Bioprospect. 2024 Jan 16;14(1):10. doi: 10.1007/s13659-024-00428-0 (PMC10789705; doi:10.1007/s13659-024-00428-0)
Supplement: Supplementary file 1 — Additional file SI 1: Fig. S1. Polysaccharide (CSGC) purity by high-performance gel permeation chromatography (HPGPC) profiles. The presence of 1 mg/mL CSGC monitored by HPLC-ELSD. Chromatographic conditions: sample: 1 mg/mL; chromatographic column: Shodex OHpak-SB-804 HQ 8 mm*30 cm; mobile phase: 100% water isocratic elution; Elution time: 15 min; Injection volume: 20 μL. Fig. S2. Polysaccharide (CSGs) purity by high-performance gel permeation chromatography (HPGPC) profiles. The presence of 1 mg/mL CSGs monitored by HPLC-ELSD. Chromatographic conditions: sample: 1 mg/mL; chromatographic column: Shodex OHpak-SB-804 HQ 8 mm*30 cm; mobile phase: 100% water isocratic elution; Elution time: 15 min; Injection volume: 20 μL. Fig. S3. The interactions of aqueous solution. Fig. S4. Scanning electron micrographs of CSGC and CSGs: (a-3) CSGC30; (b-3) CSGC50; (c-3) CSGC70; (d-3) CSGC90; (e-3) CSG30; (f-3) CSG50; (g-3) CSG70; and (h-3) CSG90; (3, 20000×). Table S1. SPR analysis showed that CSGS and GLUT-1 proteins had direct binding KD values [file 13659_2024_428_MOESM1_ESM.docx]

**Additional file SI**

**Interaction mechanism between** **luteoloside and** **corn silk glycans and** **the** **synergistic role in hypoglycemic activity**

Shihui Qin ^a,c 1^, Yanlang Li ^c 1^, Huiyan Shao ^c^, Yang Yu ^a^, Yina Yang ^a^, Yi Zeng ^a^, Jia Huang ^c^, Jiang-miao Hu ^c^ *, Liu Yang^b,c^*

Dr. S. Qin, Y. Yu, Y. Yang, Y. Zeng

a *College of Pharmacy, Anhui University of Chinese Medicine, Hefei, Anhui 230038, China*

Dr. L. Yang

b State Key Laboratory of Quality Research in Chinese Medicine, Macau Institute for Applied Research in Medicine and Health, Macau University of Science and Technology, Taipa, Macau 999078, China;

Dr. L. Yang, H. Shao, J. Huang, Y. Li, J. -M. Hu

c State Key Laboratory of Phytochemistry and Plant Resources in West China, and Yunnan Key Laboratory of Natural Medicinal Chemistry, Kunming Institute of Botany, Chinese Academy of Sciences, Kunming, Yunnan, 650201, China

E-mail: hujiangmiao@mail.kib.ac.cn；yangliu@mail.kib.ac.cn

* **Corresponding Author.**

E-mail address: [hujiangmiao@mail.kib.ac.cn](mailto:hujiangmiao@mail.kib.ac.cn) (J.M. Hu.); [yangliu@mail.kib.ac.cn](mailto:yangliu@mail.kib.ac.cn) (L. Yang.)

^1^ These two authors contributed equally to this work.

**Contents of Additional file SI**

[**Fig. S1** Polysaccharide (CSGC) purity by high-performance gel permeation chromatography (HPGPC) profiles. 1](#_Toc155479701)

[**Fig. S2** Polysaccharide (CSGs) purity by high-performance gel permeation chromatography (HPGPC) profiles. The presence of 1 mg/mL CSGs monitored by HPLC-ELSD. Chromatographic conditions: sample: 1 mg/mL; chromatographic column: Shodex OHpak-SB-804 HQ 8 mm*30 cm; mobile phase: 100% water isocratic elution; Elution time: 15 min; Injection volume: 20 μL. 1](#_Toc155479702)

[**Fig. S3** The interactions of aqueous solution. 2](#_Toc155479703)

[**Fig. S4** Scanning electron micrographs of CSGC and CSGs.(a-3) CSGC30; (b-3) CSGC50; (c-3) CSGC70; (d-3) CSGC90; (e-3) CSG30; (f-3) CSG50; (g-3) CSG70; and (h-3) CSG90; (3, 20000×). 3](#_Toc155479704)

[**Table S1** SPR analysis showed that CSGS and GLUT-1 proteins had direct binding KD values. 3](#_Toc155479705)


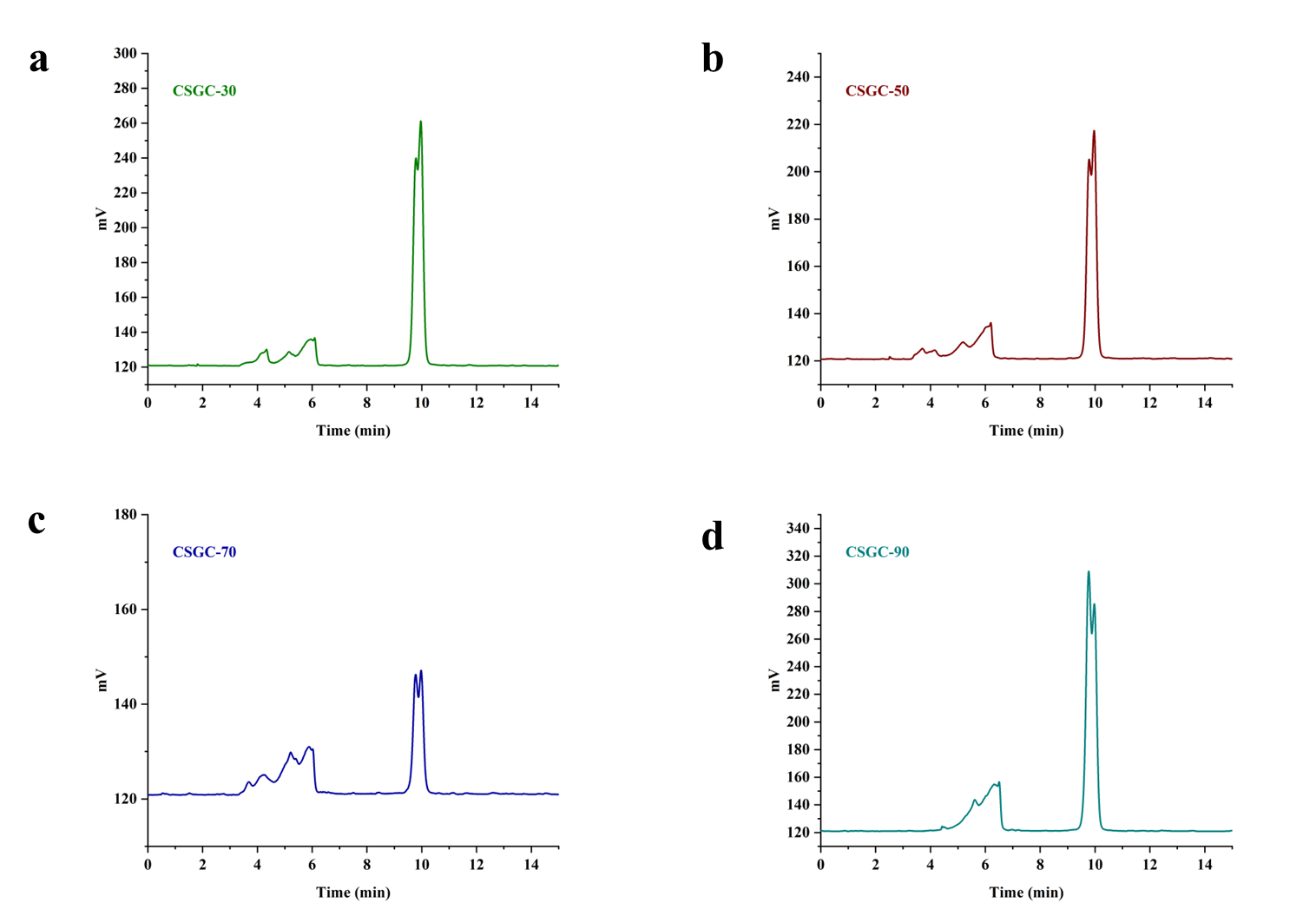


**Fig. S1** Polysaccharide (CSGC) purity by high-performance gel permeation chromatography (HPGPC) profiles.

The presence of 1 mg/mL CSGC monitored by HPLC-ELSD. Chromatographic conditions: sample: 1 mg/mL; chromatographic column: Shodex OHpak-SB-804 HQ 8 mm*30 cm; mobile phase: 100% water isocratic elution; Elution time: 15 min; Injection volume: 20 μL.


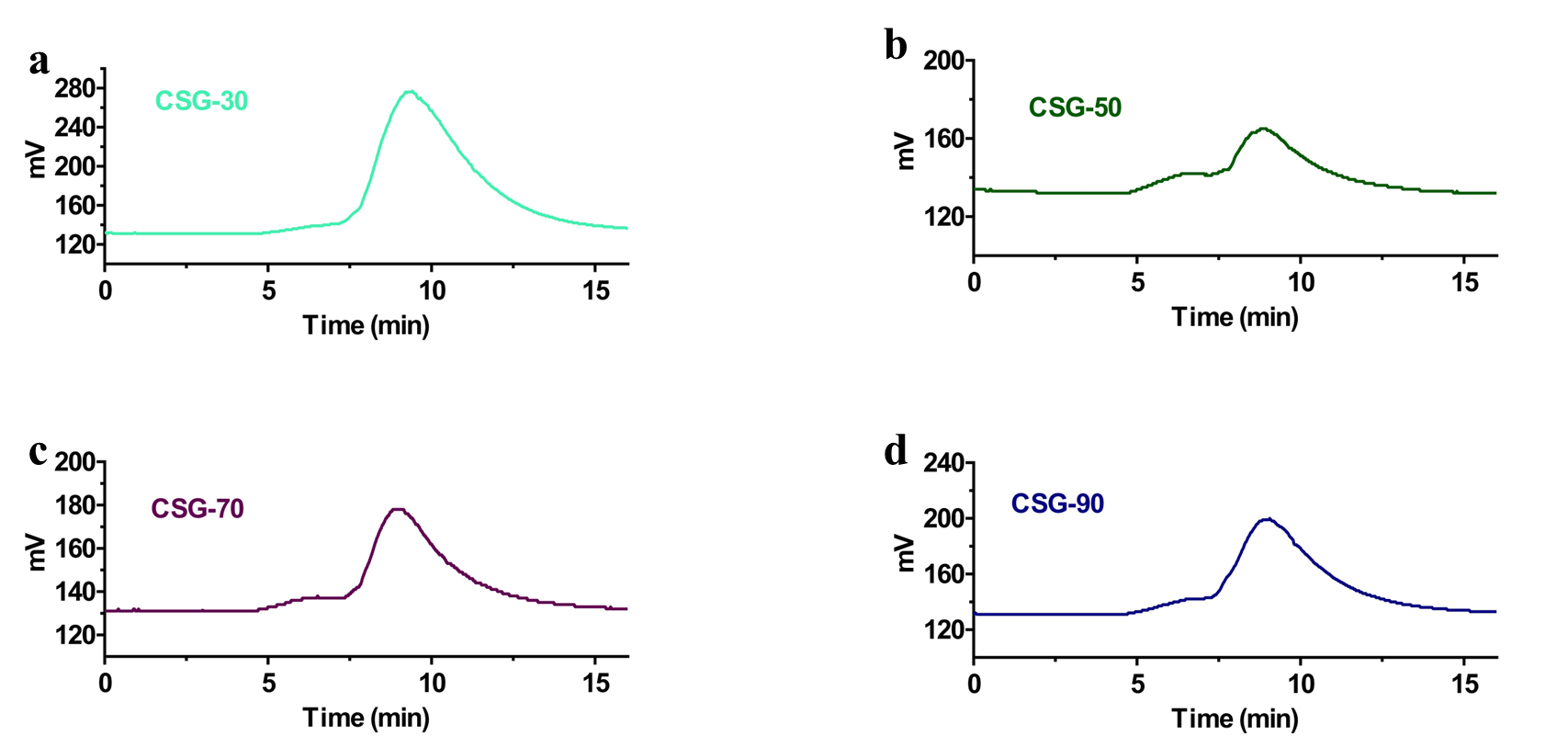


**Fig. S2** Polysaccharide (CSGs) purity by high-performance gel permeation chromatography (HPGPC) profiles. The presence of 1 mg/mL CSGs monitored by HPLC-ELSD. Chromatographic conditions: sample: 1 mg/mL; chromatographic column: Shodex OHpak-SB-804 HQ 8 mm*30 cm; mobile phase: 100% water isocratic elution; Elution time: 15 min; Injection volume: 20 μL.

**
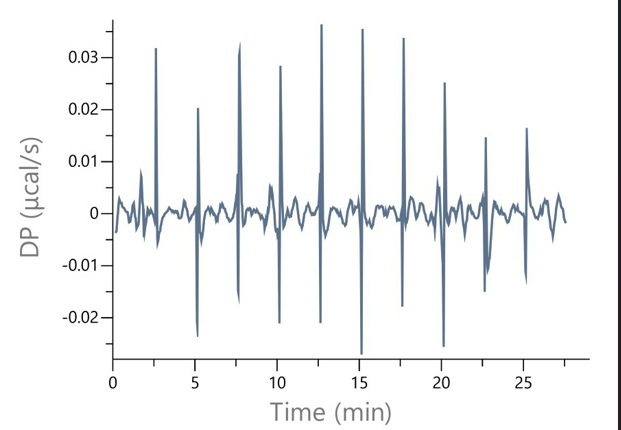
**

**Fig. S3** The interactions of aqueous solution.


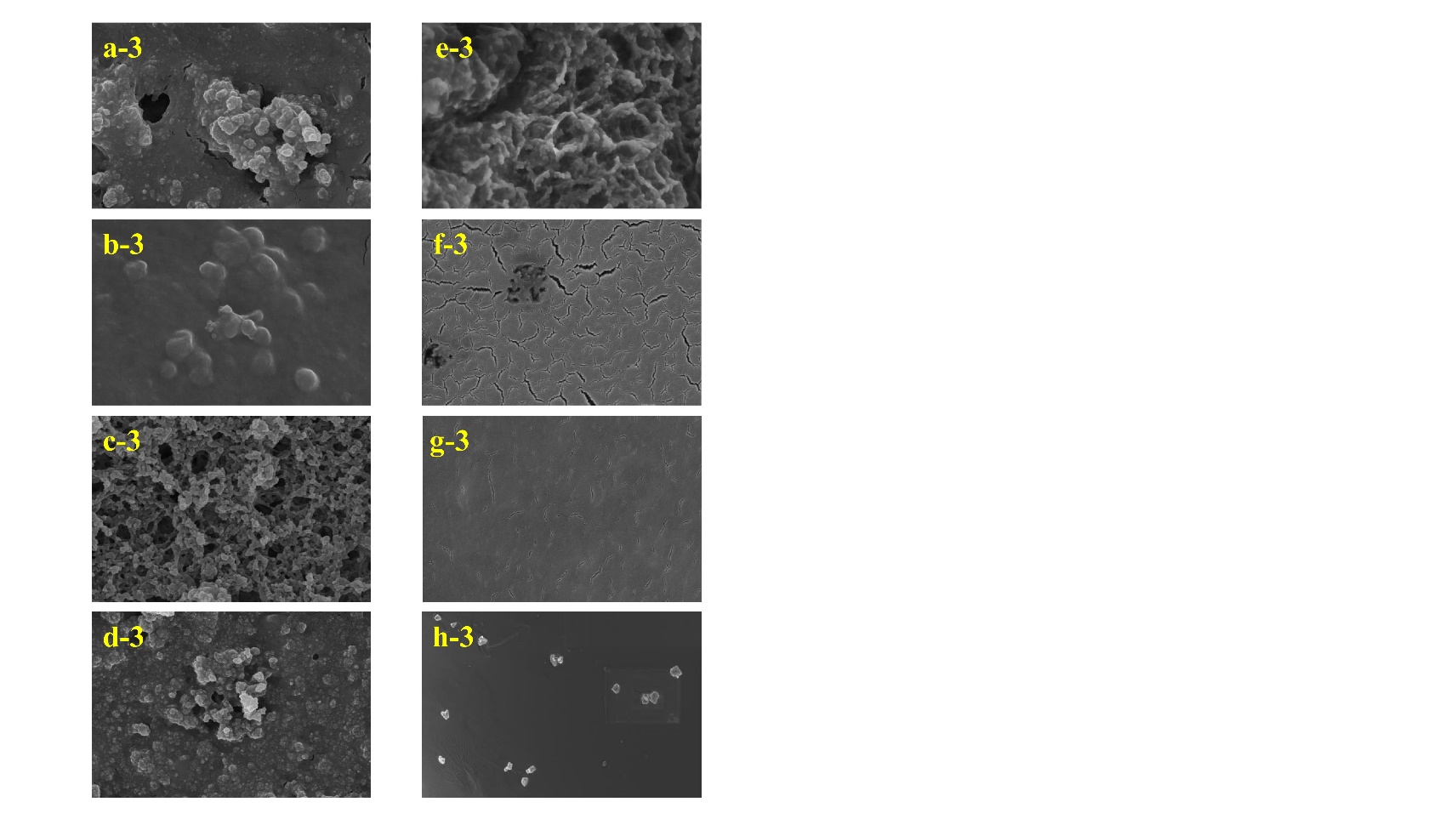


**Fig. S4** Scanning electron micrographs of CSGC and CSGs.(a-3) CSGC30; (b-3) CSGC50; (c-3) CSGC70; (d-3) CSGC90; (e-3) CSG30; (f-3) CSG50; (g-3) CSG70; and (h-3) CSG90; (3, 20000×).

**Table S1** SPR analysis showed that CSGS and GLUT-1 proteins had direct binding KD values.

| Sample | KD (GLUT-1) |
| --- | --- |
| LUT | 2.2×10^2^ |
| CSG30 | 1.65×10^-2^ |
| CSG50 | 1.72×10^-5^ |
| CSG70 | 16.88 |
| CSG90 | 4.30×10^-6^ |
| LUT+CSG30 | 1.70×10^-4^ |
| LUT+CSG50 | 1.73×10^-4^ |
| LUT+CSG70 | 2.02×10^-4^ |
| LUT+CSG90 | 1.17× 10^-2^ |
